# Perceptual-cognitive skill and the in situ performance of soccer players

**DOI:** 10.1080/17470218.2016.1255236

**Published:** 2016-11-21

**Authors:** Mariëtte J. J. van Maarseveen, Raôul R. D. Oudejans, David L. Mann, Geert J. P. Savelsbergh

**Affiliations:** 1 Department of Human Movement Sciences, Vrije Universiteit Amsterdam, MOVE Research Institute Amsterdam, Amsterdam, The Netherlands; 2 Faculty of Sports and Nutrition, Amsterdam University of Applied Sciences, Amsterdam, The Netherlands

**Keywords:** Anticipation, Decision making, Gaze behaviour, In situ performance, Pattern recall

## Abstract

Many studies have shown that experts possess better perceptual-cognitive skills than novices (e.g., in anticipation, decision making, pattern recall), but it remains unclear whether a relationship exists between performance on those tests of perceptual-cognitive skill and actual on-field performance. In this study, we assessed the in situ performance of skilled soccer players and related the outcomes to measures of anticipation, decision making, and pattern recall. In addition, we examined gaze behaviour when performing the perceptual-cognitive tests to better understand whether the underlying processes were related when those perceptual-cognitive tasks were performed. The results revealed that on-field performance could not be predicted on the basis of performance on the perceptual-cognitive tests. Moreover, there were no strong correlations between the level of performance on the different tests. The analysis of gaze behaviour revealed differences in search rate, fixation duration, fixation order, gaze entropy, and percentage viewing time when performing the test of pattern recall, suggesting that it is driven by different processes to those used for anticipation and decision making. Altogether, the results suggest that the perceptual-cognitive tests may not be as strong determinants of actual performance as may have previously been assumed.

Perceptual-cognitive skills such as anticipation and decision making are crucial for successful performance in many complex dynamic motor tasks. For example, in aviation, the military, when driving a car, and in sport, the ability to pick up visual information and to select and execute an appropriate action is key to high-level performance (Williams & Ericsson, 2005; Williams, Ford, Eccles, & Ward, 2011). Sports offer a unique, dynamic, and time-constrained environment in which perceptual-cognitive skills can be examined. In team sports, like soccer, expert performance means choosing the correct action at the correct moment and performing that course of action efficiently and consistently throughout a match (Baker, Cote, & Abernethy, 2003; Gréhaighne, Godbout, & Bouthier, 2001). The ability to measure the level of performance on these perceptual-cognitive tasks is crucial to better understand expert performance, and to identify the factors and underlying processes that mediate successful performance (Williams & Ericsson, 2005). Accurate measures of perceptual-cognitive skill could be used, for instance, for the purposes of talent identification and development, and to determine the efficacy of training interventions designed to improve performance. However, it remains unclear what might be the best way to measure perceptual-cognitive skill to accurately reflect the demands of actual on-field performance (Mann & Savelsbergh, 2015; Pinder, Headrick, & Oudejans, 2015; Williams & Ericsson, 2005), and this remains a significant barrier for scientists and practitioners who wish to better understand and improve high-level performance in dynamic motor tasks.

Perceptual-cognitive skill as it is performed in motor tasks has typically been measured using simplified video-based tests in which participants do not move, but instead indicate their preferred action or response from a variety of options either verbally or by way of a button press (e.g., Abernethy & Russell, 1987; Gorman, Abernethy, & Farrow, 2012; Savelsbergh, Williams, Van der Kamp, & Ward, 2002). Using this method, clear differences have been revealed between experts and novices, and sometimes differences are studied within groups to discriminate those with relatively high and low levels of perceptual-cognitive skill (e.g., Savelsbergh, van der Kamp, Williams, & Ward, 2005). Skilled performers are consistently found to be superior on a variety of perceptual-cognitive tasks including those designed to test (a) *anticipation*, the ability to predict the outcome of another person’s action on the basis of the pick-up of early visual information (e.g., Abernethy & Russell, 1987; Jones & Miles, 1978; Savelsbergh et al., 2002; Williams & Ward, 2007); (b) *decision making*, the ability to select the best possible option from a variety of alternatives (e.g., Helsen & Pauwels, 1993; Vaeyens, Lenoir, Williams, Mazyn, & Philippaerts, 2007); and (c) *pattern recall*, the ability to recall previously seen patterns of play (e.g., Allard, Graham, & Paarsalu, 1980; Gorman et al., 2012; Van Maarseveen, Oudejans, & Savelsbergh, 2015). In addition, differences in gaze behaviour are often reported when these tasks are performed, generally showing that experts use fewer fixations of longer duration than novices (e.g., Mann, Williams, Ward, & Janelle, 2007), a finding that has been interpreted to suggest that experts use a more efficient search strategy when performing these tasks (Helsen & Pauwels, 1993).

Although the traditional video-based perceptual-cognitive skill tests offer a significant advantage in terms of their methodological rigour and control, it remains unclear how well these tests might accurately represent the on-field performance they are designed to sample (Mann & Savelsbergh, 2015; Pinder et al., 2015; Williams & Ericsson, 2005). Recently, significant differences have been found in both movement and visual behaviour when comparing performance on traditional video-based tests with contexts that are likely to be more representative of the participants’ performance environment (Dicks, Button, & Davids, 2010; Pinder et al., 2015). For example, Dicks et al. (2010) showed that when compared to responding to a video simulation, soccer goalkeepers made more penalty saves and fixated earlier on the ball and for longer periods of time in an in situ condition where actual interception was required. Similarly, Mann, Abernethy, and Farrow (2010) found that anticipation skill increased when participants were required to make an actual movement rather than a simple verbal response when anticipating the direction of a cricket ball. In support, a meta-analysis of perceptual-cognitive skill in sport has shown that expertise effects are most apparent when participants have to perform genuine actions under in situ task constraints rather than performing simplified responses in less representative conditions (Mann et al., 2007; Travassos et al., 2013).

The decoupling of perception and action provides a clear distinction between task designs in which participants are required to make actual movements (an *action* response) and those in which participants generally respond verbally or by a simplified movement like a button-press (generally considered to be *perceptual* responses). The two-visual system model of Milner and Goodale (1995) claims that action and perception rely on two neuro-anatomically separate visual pathways within the brain: The ventral “vision-for-perception” stream is thought to be used for perceiving what action a situation affords, and the dorsal “vision-for-action” stream for the visual guidance of that action. In a persuasive position paper that examined the implications of the dual pathway model for research on *anticipation*, Van der Kamp, Rivas, Van Doorn, and Savelsbergh (2008) suggested that much of the previous anticipation research had largely examined only the role of the ventral pathway because those studies had relied on video-based tests in which no actual movement had to be made. By excluding action from the participant response, Van der Kamp et al. (2008) claimed that most existing studies overlook the contribution of the dorsal system that is most likely to be relied on during actual performance. This distinction provides reason to believe that video-based tests of anticipation are likely to under-represent (or even misrepresent) the true ability of skilled performers when performing in an actual performance setting (Dicks, Davids, & Button, 2009; Mann et al., 2007). The same could also be said for tests of decision making, where participants must perceive the situation in order to decide an appropriate action to perform. Therefore, it could be that decision making is also likely to be affected by the absence of an action response in the same way that tests of anticipation might be. In support, Oudejans, Michaels, Van Dort, and Frissen (1996) examined safe road-crossing behaviour and showed that more accurate decisions were made when people walked towards the road than if they were standing still and making the same decision. However, given that the recall of briefly presented patterns of play is rarely required in the natural performance environment (Gorman, Abernethy, & Farrow, 2013; Williams & Ericsson, 2005) and that doing so is unlikely to be coupled to an action, it could be that the impact that absence of action would have on a test of pattern recall might be less pronounced than it would be for tests of anticipation and decision making. The test of pattern recall is likely to be a highly perceptual test for which there might not be an equivalent test that would rely on a motor response.

The degree to which different perceptual-cognitive skills are related is an important topic of recent debate (Farrow, McCrae, Gross, & Abernethy, 2010). In particular, it has been suggested that pattern recall may serve a functional role for facilitating anticipation and decision making. It has been claimed that athletes may use the locations of players to anticipate the next state of the pattern of play and to make an appropriate decision in response to this evolving pattern (Farrow et al., 2010; Gorman et al., 2012, 2013; Williams & Davids, 1995). This is a significant issue as it helps to reveal whether pattern recall, anticipation, and decision making are independent skills that should be acquired separately, or whether they are all related and underpinned by one underlying skill and thus similar cognitive processing (Gorman, Abernethy, & Farrow, 2015; North, Williams, Hodges, Ward, & Ericsson, 2009). Moreover, from a practical perspective, there would be no need to administer multiple tests if they were to be assessing the same underlying attribute. The majority of research to date has examined performance on the different tests of perceptual-cognitive skill independently (Williams & Ward, 2007), with only a few studies having searched for any relationship between those skills. One exception was a study by Farrow et al. (2010) who examined correlations between the anticipation and pattern recall skill of expert, intermediate, and novice rugby union players in line-outs. They found that pattern recall skill accounted for 40% of the variance in the anticipation task; however, when the level of expertise was accounted for they found that the correlation between anticipation and pattern recall remained for the intermediate and novice players only, and not for the experts. Farrow et al. consequently suggested that lesser skilled players use pattern recall when attempting to anticipate an evolving pattern, but for experts the contribution of pattern recall is diminished, and the anticipation task is processed in a different manner.

One possible way to better understand the degree to which different tests of perceptual-cognitive skill might be related, and thereby the underlying processes relied on when performing those tasks, is through the examination of gaze behaviour (Williams & Ericsson, 2005). In 1967, Yarbus first showed that gaze behaviour changes as a result of task requirements, even when the same visual stimulus is viewed (in that case stationary images). Similar results have been found within the sports domain; for example, Gorman et al. (2015) found differences in the gaze strategies of skilled basketball players when watching the same video footage for the purposes of decision making and pattern recall, and North et al. (2009) found differences in the gaze of soccer players when watching video clips for the purposes of pattern recognition and anticipation. Differences in gaze behaviour between the various tests has been interpreted to provide support for the idea that different processes underpin these contrasting perceptual-cognitive skills (North et al., 2009).

To better understand and improve high-level performance in dynamic motor tasks, the fundamental question of interest in establishing appropriate tests of perceptual-cognitive skill is whether performance on those tests predicts on-field performance. Existing studies have used the expert–novice comparison to show differences between skill levels, and assumed that those perceptual-cognitive skills for which there are differences must comprise an important element of expertise. It could be that some perceptual–cognitive skills are more related to the actual performance on the field than others, and this could depend on how well the separate tests reflect the processes that are needed for actual actions on the field. Therefore, in some studies, the relative weight of factors contributing to skilled performance have been examined—for example, Ward and Williams (2003) assessed young soccer players using a multidimensional battery of tests and found that anticipation and the use of situational probabilities (i.e., expectations of what is likely to happen next) were the best discriminating factors across the different skill groups. However, this expert–novice approach falls short of being able to provide direct evidence that performance on those tests is related to on-field performance. Rather, superior performance could be a consequence of experience in the game instead of being a contributing factor to expertise. As a result the relationship between these perceptual-cognitive skills and actual performance remains unclear (Ericsson, Patel, & Kintsch, 2000; Ericsson & Smith, 1991).

In the current study, we sought to examine how well performance in a complex time-constrained motor task could be predicted using representative tests of perceptual-cognitive skill. To do so we assessed the in situ performance of young talented soccer players in a small-sided soccer game and related it to their performance on separate tests of anticipation, decision making, and pattern recall. Moreover we sought to determine the degree to which the three tests of perceptual-cognitive skill were related by exploring the correlations between the tests and the similarity of the gaze of participants when performing those tasks. If performance on the tests of perceptual-cognitive skill were to be highly predictive of on-field performance then strong within-group correlations should be found between the measures of perceptual-cognitive skill and individual performance in the small-sided games. Moreover, if performance on the three tests of perceptual-cognitive skills were to be highly correlated with each other, then similarities in gaze behaviour when performing those tasks would be expected. Instead, if the degree to which the skills were to overlap would be low then significant differences in gaze would be expected when participants were performing those tasks. Insight into the degree to which the perceptual-cognitive skills overlap and how well these tests represent in situ performance helps to reveal whether those skills are underpinned by different cognitive processes, and may facilitate the development of an accurate method to evaluate performance in complex time-constrained motor tasks.

## Experimental study

### Method

#### Participants

Twenty-two highly talented female soccer players from the national soccer talent team participated in the study (*M*_age_ = 16.3 years, *SD* = 1.1). They trained about 15 to 20 hours a week and played in a high-level competition for men under 14 years of age and had an average of 9.8 years (*SD* = 2.3) of soccer experience. The experiment was approved by the local ethics committee of the research institute, and all participants gave their written informed consent prior to the experiment; parental consent was provided for players younger than 18 years.

#### In situ test

**Figure 1.**
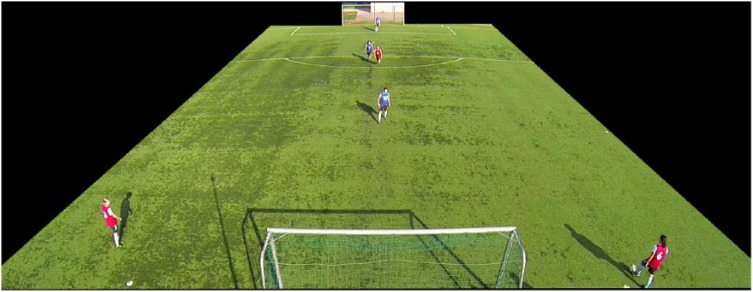
Snapshot of video clip of the small-sided game. Players are located at their specific starting positions. To view this figure in colour, please visit the online version of this Journal.

The in situ test was identical to the one described by Van Maarseveen, Oudejans, and Savelsbergh (in press). The test comprised 3 versus 3 small-sided games (i.e., three attackers vs. two defenders and a goalkeeper) because these games are considered to comprise the basics of the game of soccer according to the Dutch Royal Soccer Association (KNVB; Dokter, 1993), and many more behavioural observations are possible in a given period of time when compared to an 11 versus 11 game (Davids, Araújo, Correia, & Vilar, 2013). Games were played on a field of 40 m × 25 m (field dimensions were advised by the head coach). The six players started at specific locations (Figure 1) and played according to the official soccer rules, including the use of the offside rule. In each test session participants played five times in each of the playing positions. In total, eight test sessions were conducted across 4.5 months, resulting in a total of 733 trials, an average of 34 trials per participant per playing position. The test sessions were video recorded using a Go-Pro Hero 3 camera (Black Edition, resolution 1920 × 1080 pixels, 30 Hz; Go-Pro, USA) that was fixed on a 6.5-m high platform (Showtec LTB-200/6 Lifting Tower, The Netherlands) behind the goal being defended by the attacking team.

The performance of the participants was assessed using the notational system designed by Van Maarseveen et al. (in press). In this system at any one point in time each player has one of three roles: attacker with ball, attacker without ball, and defender. For each role, the possible actions and outcomes as well as the a priori determined number of points a player earns when performing that action (and the consequent outcome) were determined by two experts with over 25 years of experience in coaching soccer at a national and international level (see Table 1). For example, when an attacker with the ball shoots at the goal but the shot is saved by the goalkeeper, the player earns nine points. A slightly different approach was used to evaluate the positioning of a player, with the duration of time that the player was open or marked being registered and used to calculate the percentage of time a player spent in each of the positioning categories (“Open, own half, centre”; “Open, own half, side”; “Open, opponents’ half, centre”; “Open, opponents’ half, side”; “Marked”). The overall score for positioning was calculated by multiplying the percentage of time in each category by the number of points allocated to that specific category (Table 1). For example, when a player was open in her own half, in the centre of the field, for 30% of the total time, then this player received 0.30 × 2 = 0.6 points for this positioning category (for more details see Van Maarseveen et al., in press).

**Table 1.** Actions, outcomes, definitions, and allocation of points of notational system.

Role	Action	Outcome	Definition	Points
*Attacker with ball*				
	Shooting		The attacker shoots at goal and . . .	
		Goal	. . . scores	12
		Blocked by defender	. . . the shot is blocked by a defender	6
		Saved by goalkeeper	. . . the shot is saved by the goalkeeper	9
		Post/crossbar	. . . the ball hits the post or crossbar	9
		Wide/over	. . . the ball goes wide or over the goal (within 1 m)	6
		Far wide/far over	. . . the ball goes far wide or over the goal (more than 1 m)	0
	Passing		The attacker passes the ball . . .	
		Successful, towards teammate in promising position	. . . and a teammate in a promising position receives the ball	5
		Successful, forward	. . . forward to a teammate who receives the ball	2
		Successful, backward	. . . sideways or backward to a teammate who receives the ball	1
		Intercepted	. . . and a defender or goalkeeper intercepts the ball	0
		Offside	. . . towards a teammate in offside position	0
		Out of play	. . . out of play	0
	Dribbling		The attacker moves the ball, after receiving and prior to passing/shooting, (without a near defender) and . . .	
		Maintain ball possession, towards promising position	. . . the attacker maintains ball possession and moves towards a promising position	5
		Maintain ball possession, forward	. . . the attacker maintains ball possession and moves forwards	2
		Maintain ball possession, to the side or backward	. . . the attacker maintains ball possession and moves to the side or backwards	1
		Ball possession lost	. . . the attackers loses ball possession	0
	Offensive 1:1 duel		The attacker with ball and defender approach within 1 m, the defender is next to or in front of the attacker, and . . .	
		Attacker wins and overtakes	. . . the attacker wins the duel and overtakes the defender	5
		Attacker retains ball possession but goes back	. . . the attacker maintains ball possession but does not overtake the defender	3
		Defender plays ball out of play	. . . the defender plays the ball out of play	2
		Defender wins ball possession and can continue directly	. . . the defender conquers ball possession and is able to continue to play immediately	0
		Defender wins ball possession but cannot continue directly	. . . the defender conquers ball possession and is not able to continue to play immediately	0
	Receiving		The attacker receives the ball and . . .	
		Under control	. . . controls it	1
		Out of control	. . . does not control it	0
	Foul		The attackers makes a foul	0
*Attacker without ball*				
	Running action		The attacker off the ball accelerates or moves in another direction than the flow of the game and . . .	
		Defender follows, creating more space for ball carrier	. . . a defender follows the attacker, hereby creating more space for the ball carrier	2
		Got open on own half	. . . the attacker gets open on his own half of the playing field	2
		Got open on opponent’s half	. . . the attacker gets open on the opponents’ half of the playing field	4
		Wrong direction/timing	. . . the attacker does not get open and the defender does not follow him	0
	Offside		The attacker is in offside position	0
	In promising position		The attacker is in a promising position—that is, inside the penalty box, and a 2-m-wide line from the attacker towards the goal is open	7
	Foul		The attacker off the ball makes a foul	0
	Positioning		A 1-m-wide line from the ball carrier to the attacker off the ball is . . .	
		Open, own half, centre	. . . open, and the attacker off the ball is on his own half of the field and in the centre	2
		Open, own half, side	. . . open, and the attacker off the ball is on his own half of the field and at the side	1
		Open, opponents’ half, centre	. . . open, and the attacker off the ball is on the opponents’ half of the field and in the centre	5
		Open, opponents’ half, side	. . . open, and the attacker off the ball is on the opponents’ half of the field and at the side	3
		Marked	. . . marked by a defender	0
*Defender*				
	Defensive 1:1 duel		The defender and attacker with ball approach within 1 m, the defender is next to or in front of the attacker, and . . .	
		Attacker wins and overtakes	. . . the attacker wins the duel and overtakes the defender	0
		Attacker retains ball possession but goes back	. . . the attacker maintains ball possession but does not overtake the defender	2
		Defender plays ball out of play	. . . the defender plays the ball out of play	2
		Defender wins ball possession and can continue directly	. . . the defender conquers ball possession and is able to continue to play immediately	6
		Defender wins ball possession but cannot continue directly	. . . the defender conquers ball possession and is not able to continue to play immediately	4
	Defensive pressure		The defender accelerates towards the attacker with ball and approaches within 2 m (and more than 1 m) and . . .	
		Attacker goes forward	. . . the attacker with ball moves forward	0
		Attacker goes backward	. . . the attacker with ball moves to the side or backwards	3
		Towards 1:1 duel	. . . the defender approaches to within 1 m, and a 1:1 duel follows	2
	Intercepting		The defender intercepts a pass and . . .	
		Under control	. . . controls the ball	6
		No control	. . . does not control the ball	2
	Blocking shot		The defender blocks a shot at goal and . . .	
		Defender got ball possession	. . . the defender gains ball possession	5
		Defender got no ball possession	. . . the attackers maintain ball possession	2
	Offside trap		The last defender steps forward to put an attacker offside and . . .	
		Well executed	. . . the defender wins ball possession due to offside	3
		Not well executed	. . . the timing is not correct, and thus the attackers maintain ball possession	−3
	Foul		The defender makes a foul . . .	
		Inside penalty area	. . . inside the penalty area	−9
		Own half	. . . on his own half	−6
		Opponent’s half	. . . on the opponents’ half	−3

Note: Reproduced from Van Maarseveen et al. (in press) with permission.

The video footage of the in situ test was analysed frame by frame so that all actions and the consequent outcomes were registered for each player on the field. Subsequently, performance scores were determined by calculating the average number of points per trial that a player received in each of the three roles, and summing those scores into an overall performance score. Van Maarseveen et al. (in press) validated the notational system on highly talented youth soccer players. Besides high content and ecological validity, they showed significant concurrent validity (i.e., correlation between the performance scores attained with the notational system and judgments of the head coach; τs > .397, *p*s < .05), construct validity (i.e., ability of the notational system to discriminate the high- and low-skilled players, *t*s > 2.505, *p*s < .05, *r*s > .69), and reasonably good intra- and inter-observer reliability (intra: mean percentage of error = 5.7%, correlation *r*s > .87, *p*s < .001; inter: mean percentage of error = 13.7%, correlation *r*s > .89, *p*s < .001, except for one category of positioning *r* = .39, *p* < .05). Two participants did not participate in the in situ test because of injury and therefore were excluded from the study.

#### Perceptual-cognitive skill tests

##### Stimulus materials

The test stimuli for the perceptual-cognitive skill tests were identical to those used by Van Maarseveen et al. (2015) and consisted of short video clips (5 to 10 seconds) of similar 3 versus 3 small-sided games to those experienced in the in situ test, but recorded one year earlier. The video images were recorded using the same camera set-up as that employed during the in situ tests—that is, an elevated camera behind the goal defended by the attacking team. The elevated filming position was used to give a good overview of the situation and to help the participants in perceiving depth (Mann, Farrow, Shuttleworth, & Hopwood, 2009). The video clips ended at a decisive moment in the game (i.e., the onset of a shot, pass, or dribble). In order to mask irrelevant distracting features (e.g., other players who did not participate), the area outside the playing field was made black using Adobe Premiere Elements 9 (see Figure 1). To ensure that the video clips contained structured game play exemplifying the participants’ level of play, two highly experienced soccer coaches (each held the highest coaching qualifications in the country and had over 25 years of coaching experience at national and international level) independently rated the video clips on a 10-point Likert-type scale (0 = completely unstructured, 10 = completely structured), and only clips rated by both coaches with scores 7 or higher were selected (cf. Gorman et al., 2012, 2013; North & Williams, 2008; North et al., 2009).

Fourteen video clips were selected and were included in three occlusion conditions in the anticipation and decision-making test: occluded at the moment of foot–ball contact, and 100 ms (3 frames) prior to and 100 ms (3 frames) after foot–ball contact, as is a common way to test anticipation and decision-making skill (cf. Williams, Davids, & Williams, 1999). For the pattern recall test the moment of occlusion is arbitrary (as long as it occurs at a moment of structured game play), and therefore only the 14 video clips occluded at the moment of foot–ball contact were used in this test. Two additional video clips were selected as familiarization trials and were used in each test.

##### Procedure

Participants performed the perceptual-cognitive skill tests while seated in front of a large screen (i.e., the distance between the participant and the screen was about 2.5 m) onto which a projector (ASK Proxima C175, resolution 1024 × 768) displayed the video clips with an image that subtended a viewing angle of approximately 23° horizontally and 18° vertically. The participants wore SensoMotoric Instruments (SMI; Teltow, Germany) Eye Tracking Glasses, a binocular eye tracking device that recorded eye movements at 24 Hz. A one-point calibration (as advised by the manufacturer) using a small cross in the centre of the screen was performed before starting each perceptual-cognitive skill test. Each test started with instructions and two familiarization trials. The test video clips were displayed in random order, and the order of the tests was counterbalanced across participants.

The video clips were displayed, and in the anticipation and decision-making tests the clips were replaced immediately afterwards with a response slide showing buttons for four possible options: shoot, dribble, pass to the left teammate, and pass to the right teammate. In the anticipation test, the participants had to select the option that they thought the ball carrier in the video clip was going to perform at the moment of occlusion, and in the decision-making test, the participants had to select what they thought was the best option for the ball carrier. In the pattern recall test, at the moment of occlusion the video clips were replaced with an image of a blank playing field. The participants were asked to recall the last seen positions of the players and the ball by dragging Xs, Os, and a small star towards the respective positions of the defenders, attackers, and the ball (see also Van Maarseveen et al., 2015). No instructions were given about the speed of response, and hence no analyses were conducted on response times.

#### Data analysis

For the decision-making test, the correct responses were determined by two highly experienced soccer coaches (taking into account the average playing level of the participants) until consensus was reached for every trial. Response accuracy was calculated by the number of correct responses divided by the number of trials, for both the decision-making and the anticipation test.

Since previous research on pattern recall tests have shown that (a) experienced athletes anticipate the locations of the players further in advance of their actual finishing point (Gorman et al., 2012; Van Maarseveen et al., 2015), and (b) the disruptive effects of the 2D perspective of the video clip should be taken into account (Van Maarseveen et al., 2015), we assessed anticipatory pattern recall scores and used two methods to correct for the perspective effects: real-world coordinates and geometric pattern features, identical to those in Van Maarseveen et al. (2015). For the real-world coordinates method, the pixel coordinates were first transformed into real-world coordinates (using Direct Linear Transformation; Abdel-Aziz & Karara, 1971), and then the spatial error of the recalled player positions was calculated for the final frame of the video clip and for 60 subsequent frames. The smallest recall error was identified and was recorded as the *anticipatory recall score*. For the geometric pattern features method, the angles between the three attackers and the angles between the three defenders were calculated and compared to the answer templates of the final frame and the 60 subsequent frames. The smallest average error across the attackers and defenders indicated the *anticipatory pattern feature* score.

Malfunctioning of the eye-tracker (e.g., calibration problems) reduced the amount of gaze behaviour data. With our main focus being to analyse differences in gaze behaviour between the three perceptual-cognitive tests, gaze behaviour data of a particular video clip were only included in the analyses if they were available for all three tests for a particular participant. This means that only video clips occluded at the moment of foot–ball contact could be included to make valid comparisons across the three test-types, as this occlusion condition was the only one used in the test of pattern recall). This resulted in a total of 264 trials (88 video clips × 3 tests) originating from 13 participants.

The gaze behaviour was analysed frame by frame for the duration of the video clips. A fixation was defined as gaze maintained on any area of the video display for a period equal to or in excess of 125 ms or three sequential frames (cf. Savelsbergh et al., 2002; Vaeyens, Lenoir, Williams, Mazyn, et al., 2007; Vaeyens, Lenoir, Williams, & Philippaerts, 2007; Williams & Davids, 1998). The gaze behaviour of 30 randomly selected trials (i.e., 11%) was recoded by the same experimenter to assess intra-rater reliability, and a second experimenter independently coded 35 random trials (i.e., 13%) to determine inter-rater reliability. The intra-rater and inter-rater reliability both indicated good to almost perfect agreements (Hallgren, 2012), κ = .86 and κ = .79, respectively.

For each of the three tests, the four commonly used dependent variables, *search rate*, *fixation duration*, *percentage viewing time*, and *fixation order*, were calculated for each trial and were then averaged to provide a mean value for each participant. Search rate was defined as the number of fixations per second, the mean fixation duration was determined per trial, and the percentage viewing time was calculated as the percentage of total viewing time spent on each of 10 areas of interest: attacker in possession of the ball (AB), attacker without ball (A), defender (D), goal keeper (GK), ball (B), field/space (F), central spot in field/space (CF), attacker with ball closely marked by defender (AB/D), attacker without ball closely marked by defender (A/D), and other (O). The fixation order referred to the search strategy that was used by the participants and was calculated for each trial as the number of times per second that participants alternated their gaze between the player in possession of the ball, some other area in the video clip, and back to the player in possession of the ball (cf. Vaeyens, Lenoir, Williams, Mazyn, et al., 2007; Vaeyens, Lenoir, Williams, & Philippaerts, 2007; Williams & Davids, 1998; Williams, Davids, Burwitz, & Williams, 1994).

To gain more insight into the visual search strategies of the participants, we analysed to what degree the gaze behaviour was structured or randomly distributed by calculating *gaze entropy* (Allsop & Gray, 2014; Button, Dicks, Haines, Barker, & Davids, 2011; Ryu, Mann, Abernethy, & Poolton, 2016) for each test for each participant. To do this, we first calculated the number of fixation transitions between the 10 areas of interest by producing a first-order transition frequency matrix of *p*(*i* to *j*), in which *i* represents the area of interest before the transition, and *j* represents the area of interest after the transition. Separate matrices were calculated for each participant and for each test, and these were converted into conditional transition probability matrices of *p*(*j*|*t*), which gives a first-order Markov process where the probability of fixating on the *j*th area of interest is calculated, given that the previous fixation was on the *i*th area of interest. Gaze entropy can then be calculated using Ellis and Stark’s (1986) equation:

$$\mathrm{Entropy}=\overset{n}{\underset{i=1}{\sum }}p(i)\left[\overset{n}{\underset{\;j=1}{\sum }}p(j|i)\log_{2}\;p(j|i)\right]$$

In which *p*(*i*) is the zero-order probability of fixating on the *i*th area of interest (based on the percentage of total viewing time towards it), *p*(*j*|*i*) is the conditional probability of viewing area of interest *j* if the previous fixation was on area *i*, and *n* is the number of areas of interest (i.e., 10 in the current study). A higher entropy value represents a greater level of randomness in the gaze behaviour.

#### Statistical analyses

We performed some manipulation checks to examine the internal validity of the perceptual-cognitive skill tests and any learning effects as a result of watching the same video clips multiple times. For both the anticipation and decision-making tests, the accuracy scores of the three occlusion conditions (i.e., −100 ms, 0 ms, and +100 ms) were subjected to a repeated measures analysis of variance (ANOVA). To analyse whether there was a learning effect due to the repeated presentation of each of the 14 clips within one test, a repeated measures ANOVA was conducted on the accuracy scores of the first, second, and third presentation of the clips within the anticipation test and decision-making test separately. In addition, the accuracy scores of participants performing a test as the first, second, or third test were compared for each perceptual-cognitive skill test using one-way ANOVAs to check whether there was any learning effect as a result of using the same video clips in all three perceptual-cognitive skill tests.

Pearson’s correlation coefficients were calculated to investigate the relationship between the performance scores in situ and in the three tests of perceptual-cognitive skill, and for any relationship between the in situ performance scores and the gaze measures on the three tests. Also, a regression analysis was performed to examine whether the in situ performance score could be predicted by the perceptual-cognitive skill test scores. Moreover, we performed a median split on the in situ performance scores and used independent samples *t*-tests to see whether there were any differences in how the best and worst performing players in situ fared on the tests of perceptual-cognitive skill, and, vice versa, we performed median splits on the performance scores of the tests of perceptual-cognitive skill and examined whether there were any differences in the in situ performance scores. Mean values for the gaze behaviour variables search rate, fixation duration, fixation order, and entropy were compared across the three perceptual-cognitive tests using separate three-way repeated measures ANOVAs. Percentage viewing time was analysed using a 10 (area of interest) × 3 (perceptual-cognitive skill test) ANOVA with repeated measures on both factors. A Greenhouse–Geisser correction was applied to the degrees of freedom when the assumption of sphericity was violated.

### Results

#### Manipulation checks

##### Occlusion

For the anticipation test, there was a significant effect of occlusion time on the accuracy scores, *F*(1.58, 33.16) = 10.351, *p* < .001, $\eta_{p}^{2}$ = .330. Pairwise comparisons revealed that the –100-ms occlusion condition was more difficult (*M* = 64.6%, *SD* = 10.5) than the 0-ms (*M* = 71.1%, *SD* = 10.2) and +100-ms (*M* = 74.0%, *SD* = 9.2) occlusion conditions (*p* < .001; *p* < .05, respectively). There was no difference between the 0-ms and +100-ms occlusion (*p* = .726). For the decision-making test, there was no significant effect of occlusion on the decision-making test scores, *F*(2, 42) = 0.554, *p* = .579, $\eta_{p}^{2}$ = .026.

##### Learning effects

No significant differences were found between the accuracy scores of the individual *video clips* that the participants saw for the first, second, or third time in the anticipation test, *F*(2, 42) = 0.319, *p* = .729, $\eta_{p}^{2}$ = .015, nor in the decision-making test, *F*(2, 42) = 1.144, *p* = .328, $\eta_{p}^{2}$ = .052. The order in which the three *tests* were presented had no impact on the results, with no significant differences found between participants who performed each test as the first, second, or third of the three tests (anticipation test, *p* = .334, $\eta_{p}^{2}$ = .109; decision-making test, *p* = .646, $\eta_{p}^{2}$ = .045; or pattern recall test expressed in real-world coordinates, *p* = .936, $\eta_{p}^{2}$ = .007, or pattern recall features, *p* = .409, $\eta_{p}^{2}$ = .090). Thus, there were no learning effects during or across the tests as a result of repetitively watching the same video clips.

#### Relationship between performance on the in situ and perceptual-cognitive skill tests

The correlations between the in situ performance score and the scores for the anticipation, decision-making, and pattern recall tests can be found in Table 2. There were no significant correlations between the in situ performance score and any of the three tests of perceptual-cognitive skill (*r*s < .262, *p*s > .265). No significant regression equation was found that could predict the in situ performance score on the basis of performance in the perceptual-cognitive skill tests, *F*(4, 15) = 1.074, *p* = .404. Furthermore, after a median split on the in situ performance scores had been performed, the performance of the high- and low-performing participants was compared on the perceptual-cognitive skill tests. There were no significant differences between the best and worst performing players on the anticipation test, *t*(18) = 0.310, *p* = .760, *d* = 0.15, decision-making test, *t*(18) = −0.882, *p* = .389, *d* = 0.42, and pattern recall test expressed in real-world coordinates, *t*(18) = 1.309, *p* = .207, *d* = 0.62, or expressed in pattern features, *t*(18) = 0.087, *p* = .932, *d* = 0.04. And vice versa, after performing median splits on the performance scores of the perceptual-cognitive skill tests, no differences were found between the best and worst performing players on the in situ test, *ts* < 0.960, *p*s > .350, *d*s < 0.46.

**Table 2.** Correlations between in situ score and anticipation, decision-making, and pattern recall scores.

	Test	1	2	3	4
1	In situ score				
2	Anticipation	.138			
3	Decision making	−.204	.017		
4	Pattern recall real world coordinates	.262	−.354	.273	
5	Pattern recall features	.079	−.085	.306	.553*

Note: 1-4 across the top equal 1-4 reported in the first column.

* *p *< .05.

The correlations between performance on the three tests of perceptual-cognitive skill can also be found in Table 2. Again there were no significant relationships between performance on any of the three tests (*r*s < .354, *p*s > .106). The only significant correlation was a predictable one between the two varieties of pattern recall score (*r* = .553, *p* < .05)—that is, the pattern recall score expressed in real-world coordinates and the pattern recall score expressed in pattern features.

The correlations between the in situ performance score and the gaze behaviour variables of the perceptual-cognitive skill tests can be found in Table 3. Again almost none of the gaze variables were significantly related to in situ performance, with the exception being a significant correlation between the in situ performance score and the percentage of time the participants watched the ball during the decision-making test (*r* = −.662, *p* < .05), indicating that participants who scored high on the in situ test watched the ball *less* during the decision-making test.

**Table 3.** Correlations between in situ score and gaze behaviour on the anticipation, decision-making, and pattern recall tests.

Gaze variable	Anticipation	Decision making	Pattern recall
Search rate	.090	.103	.350
Fixation duration	.031	.101	−.330
Fixation order	.261	−.590	.207
Entropy	.373	.468	.384
*Area of interest*			
AB	.299	.190	.342
A	−.272	−.241	−.041
D	.028	.209	−.463
GK	−.208		.104
B	−.059	−.662*	−.174
F	.068	.219	.281
CF	.074	.569	.004
A/D	.250	−.215	−.207
AB/D	−.040	−.223	−.373
O	−.267	−.030	.181

Note: Areas of interest: Attacker in possession of the ball (AB), attacker without ball (A), defender (D), goal keeper (GK), ball (B), field/space (F), central spot in field/space (CF), attacker without ball closely marked by defender (A/D), attacker with ball closely marked by defender (AB/D), and other (O).

* *p *< .05.

#### Gaze behaviour

##### Search rate

**Figure 2.**
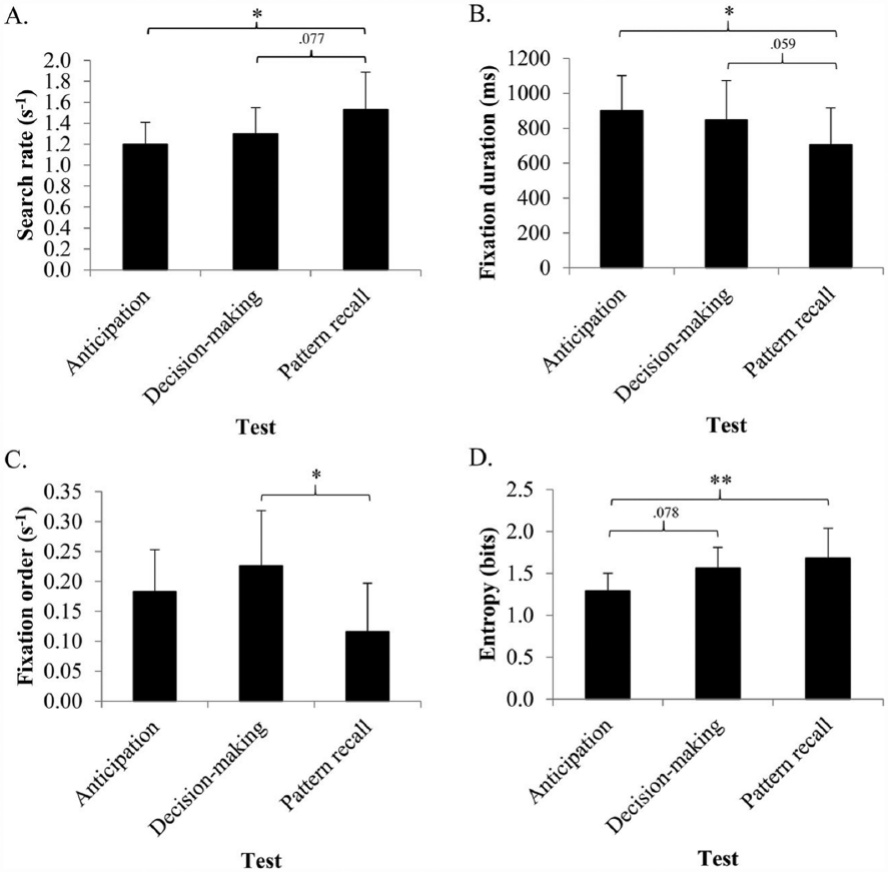
Mean search rate (A), fixation duration (B), fixation order (C), and entropy (D) for the anticipation, decision-making, and pattern recall tests. Error bars represent standard deviation; *p < .05, **p < .001.

The mean search rate (and *SD*) for each test is displayed in Figure 2A. There was a significant effect of test on the mean search rate, *F*(2, 24) = 10.021, *p* < .001, $\eta_{p}^{2}$ = .455. Post hoc Bonferroni corrected pairwise comparisons revealed that the differences were largely a result of the differences in gaze when performing the test of pattern recall. Participants made significantly more fixations per second during the pattern recall test than they did during the anticipation test (*p* < .05, *d* = 1.18), and the difference between the pattern recall test and the decision-making test approached significance (*p* = .077, *d* = 0.80). The difference between the anticipation test and the decision-making test was not significant (*p* = .184, *d* = 0.44).

##### Fixation duration

The mean fixation duration (and *SD*) for each test is displayed in Figure 2B. There was a significant effect of test on the mean fixation duration, *F*(2, 24) = 6.753, *p* < .05, $\eta_{p}^{2}$ = .360. Again the post hoc Bonferroni corrected pairwise comparisons revealed that the differences in fixation duration were largely a result of fixations of shorter duration during the test of pattern recall: The fixation duration was significantly shorter during the test of pattern recall than it was during the test of anticipation (*p* < .05, *d* = 0.98), and the differences with the decision-making test approached significance (*p* = .059, *d* = 0.68). The mean fixation durations during the decision-making test and anticipation test were not significantly different (*p* = .915, *d* = 0.26).

##### Fixation order

The mean fixation order (and *SD*) for each test is displayed in Figure 2C. There was a significant effect of test on the mean fixation order, *F*(2, 24) = 6.5510, *p* < .05, $\eta_{p}^{2}$ = .353. Post hoc Bonferroni corrected pairwise comparisons revealed the difference to be a result of significantly fewer fixation shifts (from the ball carrier to another location and back) in the test of pattern recall than in the test of decision making (*p* < .05, *d* = 1.34). There were no differences in fixation order between the decision-making and the anticipation test, and between the pattern recall test and the anticipation test (*p* = .433, *d* = 0.55; *p* = .112, *d* = 0.94, respectively).

##### Gaze entropy

The mean gaze entropy (and *SD*) for each test is displayed in Figure 2D. The test performed by the participant had a significant effect on gaze entropy, *F*(2, 24) = 8.638, *p* < .05, $\eta_{p}^{2}$ = .419. Again the difference was largely a result of a difference in the test of pattern recall, with gaze entropy being significantly higher, and thus less structured, in the test of pattern recall than it was in the test of anticipation (*p* < .001, *d* = 0.72). The difference in entropy between the tests of anticipation and decision making approached significance (*p* = .078, *d* = 0.54). The entropy during the decision-making test and the pattern recall test did not differ (*p* = .826, *d* = 0.27).

##### Percentage viewing time

**Figure 3.**
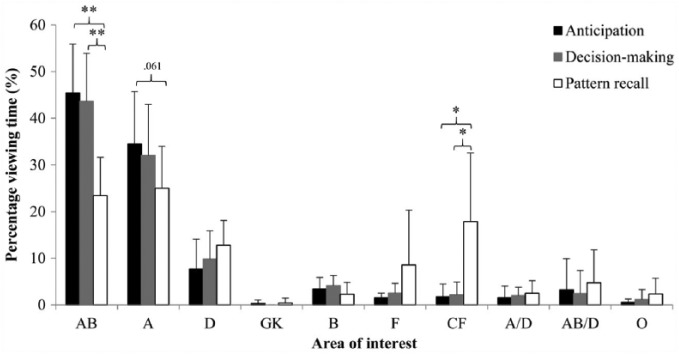
Mean percentage viewing time per area of interest for the anticipation, decision-making, and pattern recall tests. Attacker in possession of the ball (AB), attacker without ball (A), defender (D), goal keeper (GK), ball (B), field/space (F), central spot in field/space (CF), attacker without ball closely marked by defender (A/D), attacker with ball closely marked by defender (AB/D), and other (O). Error bars represent standard deviation; *p < .05, **p < .001.

The percentage viewing time per area of interest, separated for each test, is displayed in Figure 3. A significant main effect was found for area of interest, *F*(9, 108) = 94.208, *p* < .001, $\eta_{p}^{2}$ = .887, but this was overridden by a significant area of Interest × Test interaction effect, *F*(18, 216) = 11.835, *p* < .001, $\eta_{p}^{2}$ = .497. Post hoc analyses revealed that once again the differences were largely due to differences in the test of pattern recall, with participants looking less at the attacker with ball than they did during the tests of anticipation and decision making (both *p*s < .001, *d*s > 2.26). Participants looked more at a central location in the visual field during the pattern recall test than during the other tests (both *p*s < .05, *d*s > 1.55), and they tended to look less at the attackers without the ball during the pattern recall test than during the anticipation test (*p* = .061, *d* = 0.97).

## General discussion

The aim of this study was to examine how well in situ performance in a small-sided soccer game could be predicted using video-based perceptual-cognitive skill tests of anticipation, decision making, and pattern recall. We also examined the degree to which the three tests of perceptual-cognitive skill were related by exploring the correlations between the tests and the similarity of the gaze of participants when performing those tasks. The findings reveal that the in situ performance of the soccer players could not be predicted by their performance on the tests of perceptual-cognitive skill. Moreover, even a median split of the participants on the basis of their in situ performance score failed to reveal any significant differences in performance on any of the three tests of perceptual-cognitive skill, and, vice versa, median splits on the performance scores of the perceptual-cognitive skill tests failed to reveal significant differences in in situ performance scores. These findings indicate that the traditional video-based tests of anticipation, decision making, and pattern recall may not be as strong a determinant of actual performance as has been previously been assumed, and therefore caution is required at this stage in using them as conventional tests of talent in dynamic time-constrained motor tasks.

There are a number of possible explanations for the lack of any relationship between performance on the in situ test of playing ability and on the video-based tests of perceptual-cognitive skill. First, it could be that the perceptual-cognitive skills that were tested in this study are not necessary requirements of actual performance in game situations (see Ward, Williams, & Hancock, 2006; Williams & Ericsson, 2005) and consequently would not reflect the processes required for optimal on-field performance. This is possible but seems unlikely given the consistent finding of expert-related differences in performance on these types of tasks (Abernethy & Russell, 1987; Gorman et al., 2012; Helsen & Pauwels, 1993; Savelsbergh et al., 2002; Vaeyens, Lenoir, Williams, Mazyn, et al., 2007; Williams & Ward, 2007).

Second, it could be that the perceptual-cognitive skill tests are not sufficiently representative of the actual performance setting. The perceptual-cognitive skill tests are video based, and these video displays provide a less than veridical simulation of the visual information that is available in the natural performance setting (Abernethy, Gill, Parks, & Packer, 2001; Dicks et al., 2009). Projecting 3D visual information onto a 2D display causes a loss of stereoscopic depth information and a reduction in visual field and object size (Abernethy et al., 2001), and in this way it is difficult to adequately maintain the dynamic nature of the situation (Mann et al., 2007). Furthermore, the participants in this study were required to respond to the video clips using a button-press on a keyboard, meaning they were required to make a perceptual judgement and not to pick up information to control their movements or actions. According to the two-visual system model of Milner and Goodale (1995), excluding action from the participant response would diminish the contribution of the dorsal “vision-for-action” system (Van der Kamp et al., 2008). Although the implications of the distinction between perception and action have previously been shown to be particularly relevant for anticipation (Dicks et al., 2009; Mann et al., 2007; Van der Kamp et al., 2008), it seems reasonable to expect similar implications for the test of decision making (see Oudejans et al., 1996). The current study did not reveal expertise differences in any of the perceptual-cognitive tests. It is possible that expertise-related differences in performance on tests of anticipation and decision making could be found if those tests incorporated suitable movement responses. The same probably cannot be said for the test of pattern recall, as there is unlikely to be an equivalent test to the one used here that would incorporate an action.

Third, in contrast to those previous studies that have shown perceptual-cognitive skill differences between levels of expertise, the current study has shown that these video-based tests appear to be unsuitable to detect *within-group* differences between athletes of a comparable skill level. The expert–novice paradigm that is heavily relied on in studies of expertise compares the performance of participants who possess very different levels of skill. However, in a within-group comparison the more subtle differences between more successful and less successful performers within a group are compared. It could be that the video-based tests of the type used in this study are not specific enough to detect these more subtle within-group differences. Moreover, it could be that performance on the perceptual-cognitive skill tests is a by-product rather than a characteristic of expertise (though see Williams & Davids, 1995). This would suggest that caution is necessary regarding the type of scenarios and tests in which these video-based perceptual-cognitive skill tests are used.

Finally, it could be argued that the sensitivity of the in situ test of playing ability might be insufficient to pick up on any differences in skill level between the players. It could be that the measure of in situ performance is too broad, and encapsulates other factors like speed, physical fitness, or motor skills. Or it could be that the in situ measure is not sensitive enough to differentiate on-field performance. However, Van Maarseveen et al. (in press) showed that both the concurrent validity and construct validity of the in situ performance measure were good in a homogeneous skilled group of soccer players—that is, the performance scores measured using the notational analysis system significantly correlated with the subjective judgments of a highly experienced coach, and the notational analysis system demonstrated good ability to discriminate between the high- and low-skilled players within the group. Therefore, it seems unlikely that the in situ performance measure is responsible for the lack of any significant relationship between the scores of playing ability and perceptual-cognitive skill measured in this study.

This study provides some evidence to suggest that the tests of perceptual-cognitive skill are testing unique attributes that are not strongly related to each other. In particular, pattern recall skill does not appear to be the underpinning skill that supports anticipation and decision making, as has been previously suggested (e.g., Farrow et al., 2010; Gorman et al., 2012, 2013; Williams & Davids, 1995). The outcome measures for performance on the three tests of perceptual-cognitive skill provide the best evidence to suggest that all three tests are different, with there being no significant correlations between performance on any of those three tests (*p*s > .106). This is consistent with earlier studies that have found no significant correlation between the anticipation and pattern recognition skills of expert soccer players (North et al., 2009), and between the anticipation and pattern recall skills of expert rugby players (Farrow et al., 2010). Our findings highlight the need for a better understanding of the types of perceptual-cognitive skills required to attain expert performance, and whether there are other attributes that may underpin those skills. For example, future research could incorporate a test of long-term working memory to determine whether performance on any of the perceptual-cognitive tests is predicted by or related to long-term working memory (Ericsson & Kintsch, 1995).

However, in contrast to the performance measures, the evidence for unique attributes is less clear on the basis of the measurement of *gaze* when performing those tests. Based on the original findings of Yarbus (1967) and more recently on those in the sport domain (Gorman et al., 2015; North et al., 2009), we reasoned that differences in gaze behaviour when performing the tests would provide support for the idea that different underlying processes drive the way that the three different perceptual-cognitive tests are performed (Gorman et al., 2015; North et al., 2009). Gaze behaviour when performing the test of pattern recall was clearly different to that when performing the other two tests, with significant differences found for each of the five measures of gaze behaviour (search rate, fixation duration, fixation order, entropy, and percentage time spent viewing the areas of interest) when compared to the way that the tests of anticipation and/or decision making were performed. This provides strong evidence for the unique characteristic being tested when performing a test of pattern recall. During the pattern recall test the participants maintained a high search rate, presumably to scan and memorize the locations of the pattern elements as accurately as possible. They also looked more towards the centre of the field of view and tended to look less at the attackers than during the anticipation and decision-making tests, probably extracting information from outside the central area using peripheral vision to get a better overview of the pattern of play (Abernethy, 1988; Ryu, Abernethy, Mann, Poolton, & Gorman, 2013, 2015). The evidence for differences in the way that the tests were performed is less clear, though, when comparing the tests of anticipation and decision making, with no significant differences between any of the measures of gaze behaviour when those two tests were performed. There was only a borderline difference in gaze entropy (*p* = .078), providing some suggestion that gaze was more structured when performing the test of anticipation than it was when performing the test of decision making. On the basis of the measures of gaze it appears that the underlying processes responsible for anticipation and decision making might be much less distinct than that responsible when performing the test of pattern recall.

It does appear on balance, though, that participants did perform different tasks when performing the tests of anticipation and decision making. The instructions to participants in the test of anticipation were to predict what would happen next in the clip, and in the test of decision making to choose the best option available to the ball carrier at the moment of occlusion. It is possible, though, that the participants completed the anticipation test as they would the decision-making test, or, vice versa, completed the decision-making test as they would a test of anticipation. Participants chose the same response on the tests of anticipation and decision making in only 65% of cases (*SD* = 10%), providing some suggestion that the tasks were done differently (participants chose between four alternatives, and therefore the likelihood of identical answers was 25% by chance). However, much stronger evidence that the tests were performed in a unique fashion was found in the lack of correlation between the test scores for anticipation and decision making, and by the fact that, as expected, we found a significant effect of occlusion condition on the accuracy scores in the anticipation test, meaning that providing the participants with more information (i.e., a later occlusion condition) resulted in better accuracy scores, whereas in the decision-making tests we did not find an effect of occlusion condition. Thus in the decision-making test, providing the participants with more information did not result in better accuracy scores, indicating that they did not anticipate in the decision-making test. Overall, this implies that the participants approached these two tests differently and that these tests did not measure the same quality.

The findings of the present study highlight that perceptual-cognitive skill tests in their current form might not be sufficiently representative of on-field performance to reliably test for differences in skill between players of dynamic ball sports. Despite the findings of earlier studies that have shown video-based tests to be sensitive enough to pick up on group-based differences in skill, at present they seem to be less reliable for detecting within-group differences. Therefore, the findings question the suitability of video-based perceptual-cognitive skill tests for studying perceptual-motor expertise (see Dicks et al., 2010), and this suggests that caution is warranted when using these tests for talent identification or to evaluate the effectiveness of interventions. Alternatives to the paradigms employed in traditional laboratory studies have been provided by recent technological advances such as mobile eye tracking devices (Van Maarseveen et al., 2016; Pluijms, Cañal-Bruland, Kats, & Savelsbergh, 2013), event-related visual occlusion goggles (Mann et al., 2010; Oudejans, van de Langenberg, & Hutter, 2002), and virtual reality (Bideau et al., 2010; Correia, Araújo, Cummins, & Craig, 2012). In order to accurately capture the perceptual-motor performances of athletes, we suggest using in situ research designs so that the task constraints represent as accurately as possible the natural performance setting of the athlete and actual movement responses are required.

## Conclusion

Our results show that the on-field performance of talented soccer players is not predicted by performance on a common set of tests of perceptual-cognitive skill. The test of pattern recall appears to be driven by a different underlying process from that used when performing tests of anticipation and decision making, with the results of the test of pattern recall being unrelated to those of the other two tests and relying on significantly different gaze behaviour. Although performance on the test of anticipation is unrelated to that on the test of decision making, gaze behaviour remains largely unchanged on the two tests providing some suggestion that the underlying processes when performing those two tests are less distinct. In situ research designs may be more suitable to accurately capture the perceptual-motor performance of athletes so that the task constraints and response mode represent as accurately as possible the actual skill and context in which the athlete is engaged.
